# Twin Neonates With Bart’s Syndrome

**DOI:** 10.7759/cureus.21363

**Published:** 2022-01-18

**Authors:** Saleh Al-Gburi, Zainab Namuq

**Affiliations:** 1 Mosul Medical College, University of Mosul, Mosul, IRQ

**Keywords:** twin neonates, case report, epidermolysis bullosa, congenital absence of skin, bart’s syndrome

## Abstract

Bart’s syndrome is a combination of the following three criteria: congenital skin absence, blistering, and associated nail defects. We present a rare case of twins with Bart’s syndrome, who were born with congenital absence of skin and developed blisters on the skin and mucous membrane on the following days. Twins are identically affected, which confirms the genetic basis of the syndrome.

## Introduction

In 1966, a large family was found to have Bart’s syndrome, which consisted of one or more of the following three criteria: congenital skin absence, blistering, and accompanying nail problems [1]. It can be connected to any subtype of epidermolysis bullosa, i.e., simplex, junctional, or dystrophic; however, dominant dystrophic epidermolysis bullosa is more common [2].

Bart’s syndrome is an autosomal dominant condition despite the presence of sporadic cases [3]. The symptoms appear on the limbs as finely outlined, shining red lesions that stretch upward from the dorsal and medial portions of the foot to the thighs [4]. Any region of the skin can be affected; however, the illness is more common on portions of the body that are subjected to friction and damage, such as the feet, hands, arms, legs, and skin around the mouth [5].

It is a rare genetic skin disorder with only a few cases reported in the PubMed database to date, and to the best of our knowledge, this is the first report describing a twin case with Bart’s syndrome.

## Case presentation

A female twin pregnancy was delivered by a cesarean section at 35 weeks of gestation, with the twins weighing 2300 and 2200 g each. Apgar scores were 8 and 9 at the first and fifth minutes, respectively, for both.

The mother was aged 35 years and came from a poor rural region. She did not undergo regular prenatal follow-up and had no reported complications. She also denied having been exposed to any drugs or radiation during her pregnancy. Both parents appeared to be in good condition, with no anomalies of the skin, skin appendages, or mucous membrane.

**Figure 1:**
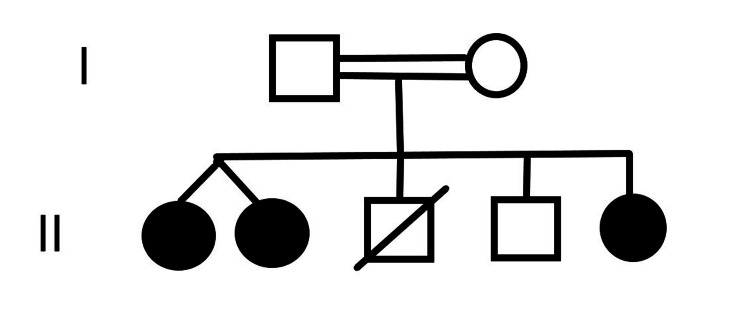
The family pedigree

Twins were the fourth pregnancy of a consanguineous marriage. The family history was remarkable, with their elder sister being affected with the disease, which was found by taking history and recollection of data from the parents. The second male child was normal, but the third child was severely affected by the disease and died five days after delivery. The family pedigree is depicted in Figure 1.

**Figure 2:**
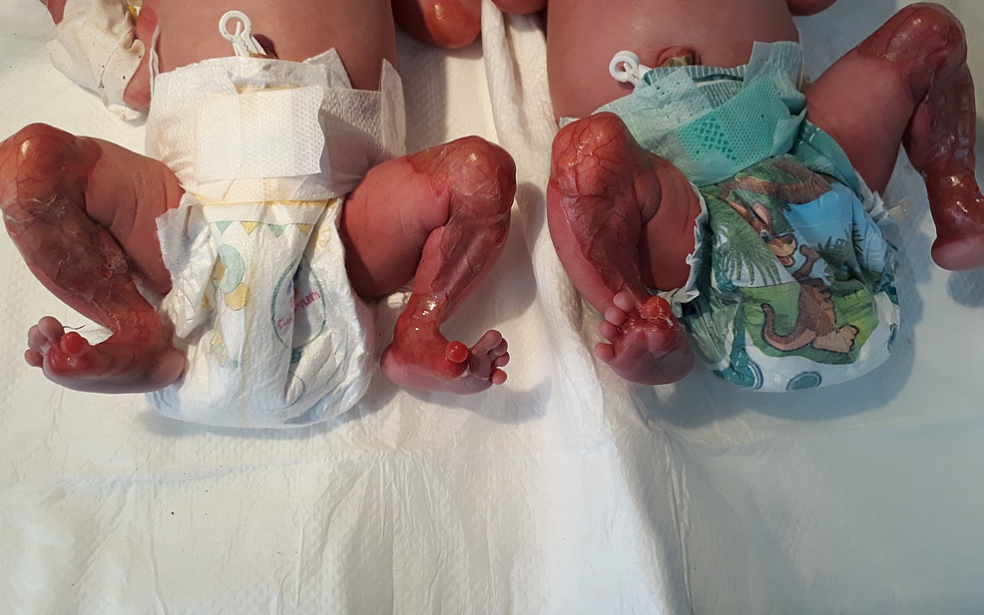
Aplasia cutis of lower extremities

**Figure 3:**
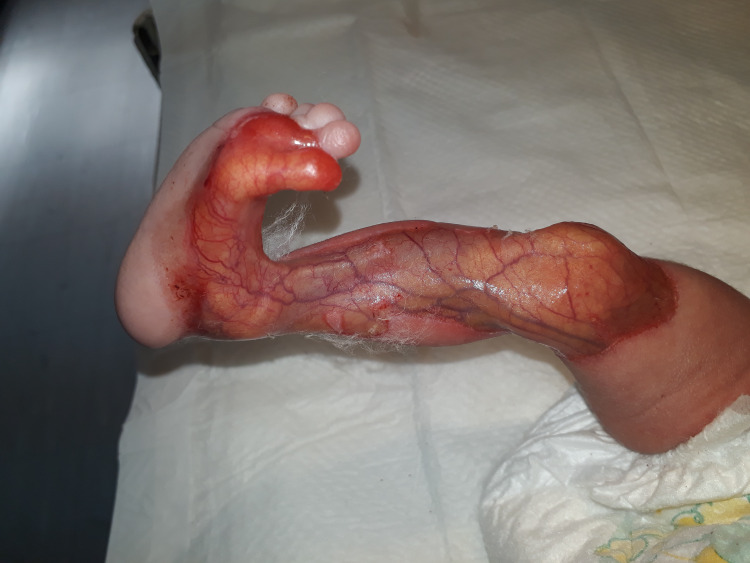
Aplasia cutis of the right lower limb

On examination, the twins had normal weight, height, and vital signs without obvious congenital deformity; however, there was a symmetrical red area without skin on the anteromedial aspect of the thighs, legs, and great toes, with easily visualized vascular structures. There was a clear separation from normal skin; aplasia cutis was diagnosed by a pediatrician (Figures 2, 3).

**Figure 4:**
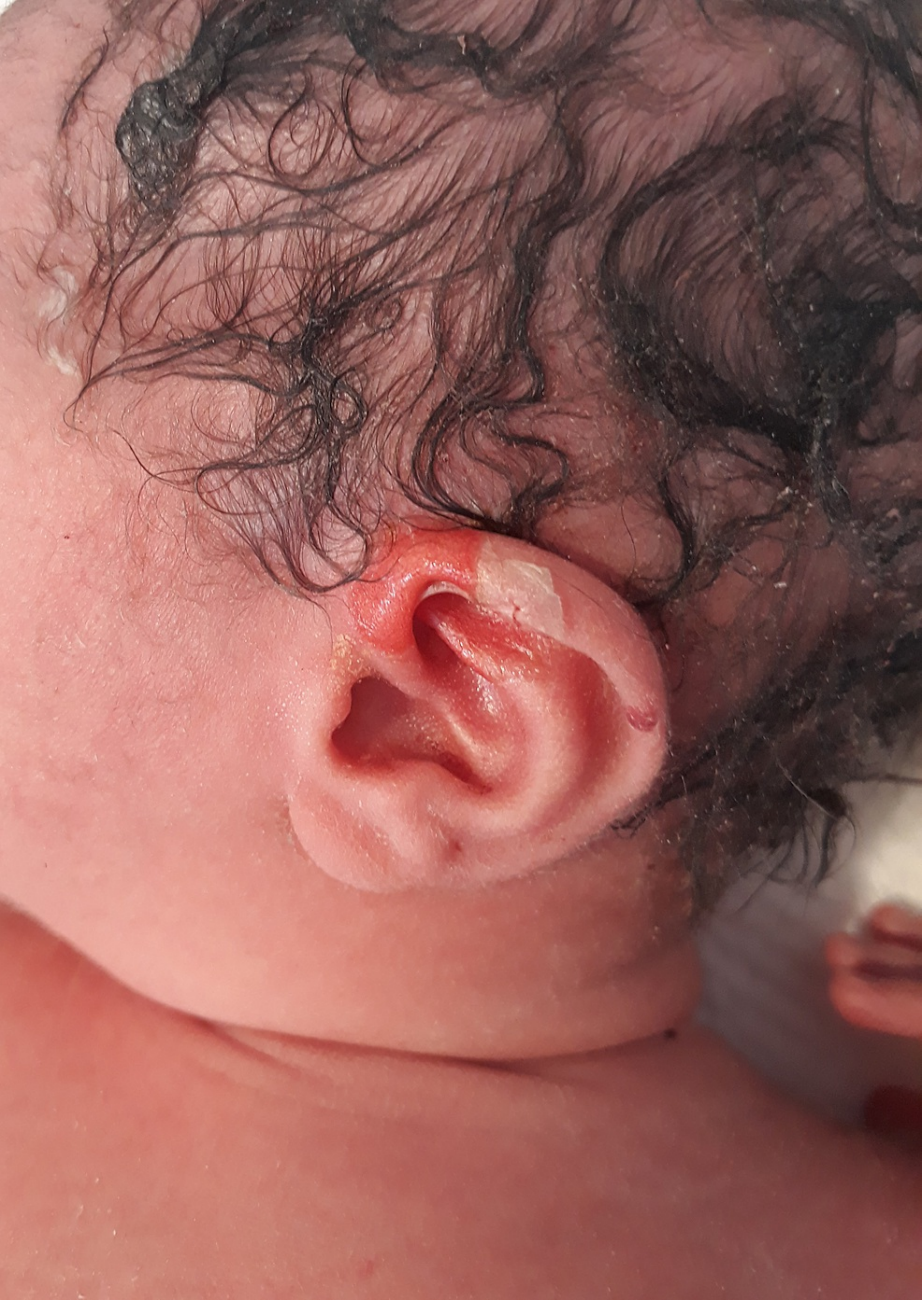
Blistering lesions on the second day

**Figure 5:**
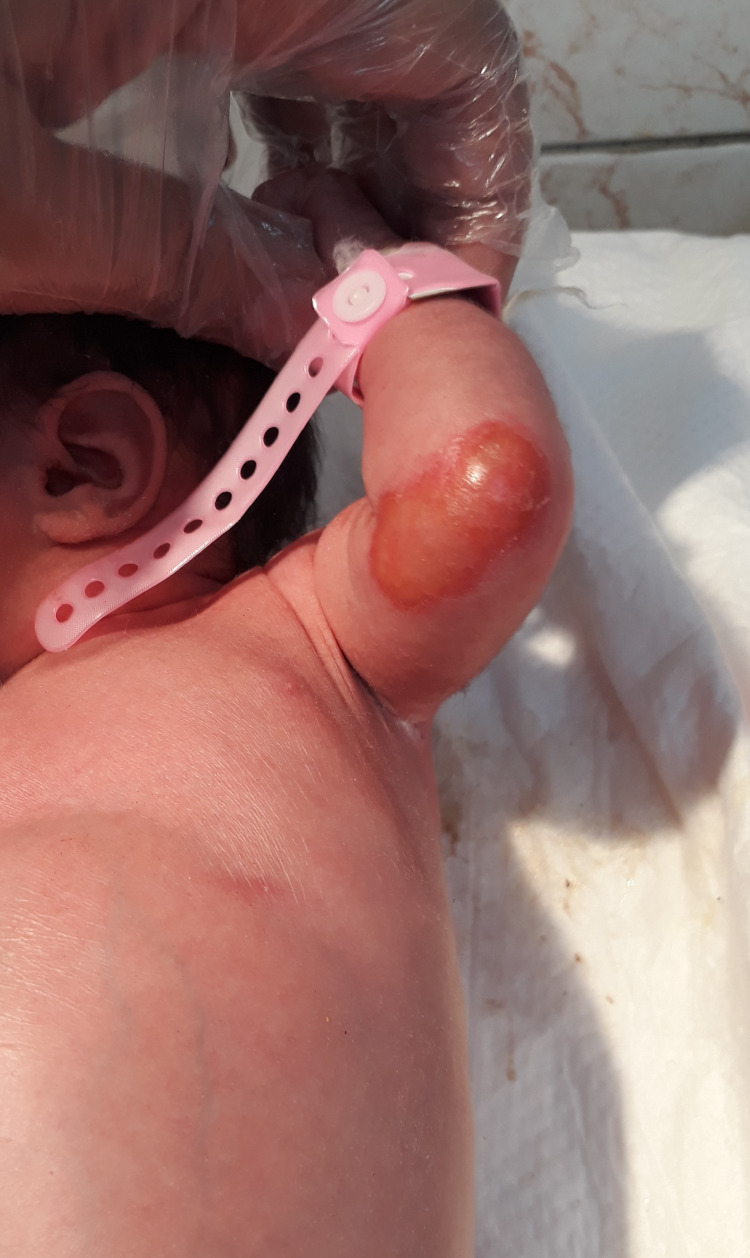
Blistering lesion over the left upper limb on the second day

The twins developed skin blisters with a gentle touch on the second day and developed oral mucosal ulceration when fed by the bottle, which was attempted because of poor mother breastfeeding (Figures 4, 5).

A clinical diagnosis of Bart’s syndrome was made based on the presence of aplasia cutis and epidermolysis bullosa. The results of complete blood count, renal function test, and liver function test were normal. Abdominal ultrasound and cardiac echocardiogram revealed normal findings. Infection-related serologic testing showed negative results. Biopsy and direct immunofluorescence were not performed because of unavailability in our hospital.

They were admitted to the neonatal intensive care unit because of infection risk. We managed the wound conservatively, and topical antibacterial treatment (2% fusidic acid cream) and nonadhesive covering were used to treat the wound. To avoid blistering, the mother was urged to keep the infants away from trauma. She was also counseled about the prognosis and outcome of the disease. The twins were referred to a plastic center in another hospital for complete management.

Patients with Bart’s syndrome generally have a fair prognosis, and one of the neonates showed improvement of the lesions, whereas the other neonate, unfortunately, died one week later because of sepsis.

## Discussion

The twins were identically affected in both lower extremities, which confirms the genetic basis of Bart’s syndrome. Ultrastructural anomalies were discovered in the anchoring fibrils in the original Bart’s syndrome family, and the disorder was linked to a region of chromosome three near the type VII collagen gene (COL7A1) [6].

In our case, the diagnosis of Bart’s syndrome was made based on the typical clinical features, which included congenital localized lack of skin across the medial portion of the lower legs, skin blistering, and nail degeneration. The clinical appearance of Bart’s syndrome is frequently used to diagnose this disease. Occasionally, a skin sample will be required to determine the type of epidermolysis bullosa, as well as genetic testing to determine the particular gene mutation, which may help us confirm the final diagnosis.

Other congenital malformations can be detected in more severe cases of Bart’s syndrome, such as pyloric atresia, choanal atresia, and corpus callosum agenesis [7,8]. However, there were no connected abnormalities in our case.

Wound pads, regular dressing, and silver sulfadiazine have been used to manage non-scalp lesions conservatively [9]. Split-thickness skin grafts, local skin flaps, and cultured epithelial autografts have been documented in the surgical treatment of aplasia cutis congenita (ACC) in non-scalp regions [10].

The degree and extent of ACC, epidermolysis bullosa subtype, accompanying abnormalities, and therapy success influence the outcome of Bart’s syndrome. Patients with Bart’s syndrome generally have a fair prognosis [11].

## Conclusions

Bart’s syndrome is a rare congenital skin disorder with a particular clinical presentation. The condition is inherited as an autosomal dominant trait with complete penetrance. It may be linked to another congenital illness, so it is essential to focus on this condition. To achieve the best outcome and avoid complications such as local infection, sepsis, hemorrhage, excessive fluid loss due to injuries, hypothermia, and electrolyte changes, care and diagnosis must be accomplished as early as possible.
